# *Mal de Río Cuarto Virus* Infection Triggers the Production of Distinctive Viral-Derived siRNA Profiles in Wheat and Its Planthopper Vector

**DOI:** 10.3389/fpls.2017.00766

**Published:** 2017-05-10

**Authors:** Luis A. de Haro, Analía D. Dumón, María F. Mattio, Evangelina Beatriz Argüello Caro, Gabriela Llauger, Diego Zavallo, Hervé Blanc, Vanesa C. Mongelli, Graciela Truol, María-Carla Saleh, Sebastián Asurmendi, Mariana del Vas

**Affiliations:** ^1^Instituto de Biotecnología, Instituto Nacional de Tecnología Agropecuaria, HurlinghamBuenos Aires, Argentina; ^2^Consejo Nacional de Investigaciones Científicas y TécnicasBuenos Aires, Argentina; ^3^Instituto de Patología Vegetal, Instituto Nacional de Tecnología AgropecuariaCórdoba, Argentina; ^4^Institut Pasteur, Viruses and RNA Interference Unit, CNRS UMR 3569Paris, France

**Keywords:** MRCV, *Fijivirus*, sRNAs, vsiRNAs, RNA silencing, wheat, planthopper, piRNAs

## Abstract

Plant reoviruses are able to multiply in gramineae plants and delphacid vectors encountering different defense strategies with unique features. This study aims to comparatively assess alterations of small RNA (sRNA) populations in both hosts upon virus infection. For this purpose, we characterized the sRNA profiles of wheat and planthopper vectors infected by Mal de Río Cuarto virus (MRCV, *Fijivirus, Reoviridae*) and quantified virus genome segments by quantitative reverse transcription PCR We provide evidence that plant and insect silencing machineries differentially recognize the viral genome, thus giving rise to distinct profiles of virus-derived small interfering RNAs (vsiRNAs). In plants, most of the virus genome segments were targeted preferentially within their upstream sequences and vsiRNAs mapped with higher density to the smaller genome segments than to the medium or larger ones. This tendency, however, was not observed in insects. In both hosts, vsiRNAs were equally derived from sense and antisense RNA strands and the differences in vsiRNAs accumulation did not correlate with mRNAs accumulation. We also established that the piwi-interacting RNA (piRNA) pathway was active in the delphacid vector but, contrary to what is observed in virus-infected mosquitoes, virus-specific piRNAs were not detected. This work contributes to the understanding of the silencing response in insect and plant hosts.

## Introduction

*Reoviridae* is a large family of viruses that can infect fungi, vertebrates, invertebrates, and plants (Attoui et al., 2011). Within this family, members of the *Phytoreovirus, Oryzavirus*, and *Fijivirus* genera can multiply in several plant species and in arthropod vectors. In plants, they cause severe diseases that threaten crop production worldwide (Lenardon et al., 1998; Dovas et al., 2004; Jiang et al., 2008; Achon and Alonso-Duenas, 2009; Wang et al., 2009; Zhou et al., 2013). Mal de Río Cuarto virus (MRCV) is a member of the genus *Fijivirus* that causes important losses in maize production in Argentina (Lenardon et al., 1998). This virus infects also wheat, barley, oat, and several grass weed species which constitute reservoirs of the virus throughout the year (Dagoberto et al., 1985; Pardina et al., 1998; Laguna et al., 2000).

MRCV virus particles have a double-shelled, icosahedral structure, and contain 10 linear double-stranded RNAs (dsRNAs) that code for six structural proteins (P1, P2, P3, P4, P8, and P10) and seven non-structural proteins (P5-1, P5-2, P6, P7-1, P7-2, P9-1, and P9-2) (Distéfano et al., 2002, 2003, 2005; Guzmán et al., 2007; Firth and Atkins, 2009). Virus progeny is produced and assembled within cytoplasmic inclusion bodies called viroplasms, which are predominately composed of P9-1 (Maroniche et al., 2010, 2012; Llauger et al., 2017). In plants, virus replication is limited to phloem tissues and causes severe symptoms such as general stunting, multiple and small ears with defective grain formation, and cell proliferations in the abaxial ribs of the leaves (Nome, 1981). In insects, fijiviruses are acquired by feeding on infected plants and transmitted in a persistent-propagative manner (Hogenhout et al., 2008; Whitfield et al., 2015). *Delphacodes kuscheli* (Hemiptera: *Delphacidae*) is the most important natural vector of MRCV (Remes Lenicov, 1985). In a closely related fijivirus, Jia et al. (2012) found that upon ingestion virus particles enter the epithelial cells of the midgut where initial replication occurs. Progeny viral particles cross the basal lamina into visceral muscle cells aided by tubules composed by P7-1 (Jia et al., 2014) and can be detected in the salivary glands approximately 17 days post-acquisition (dpa). After this latency period, for MRCV, only 20% of the viruliferous insects are able to transmit the virus to wheat (Arneodo et al., 2002). In contrast to the severe symptoms produced in plants, fijivirus infection in insects marginally alters fecundity and hatchability of the eggs, lifespan and/or feeding behavior (Arneodo et al., 2002; Tu et al., 2013; Xu H. et al., 2014).

Small RNAs (sRNAs) are a type of non-coding RNAs of 20–30 nucleotides (nt) in length that regulate various biological processes (Groszhans and Filipowicz, 2008). In plants and insects, the small interfering RNA (siRNA) pathway is critical for antiviral defense (Zvereva and Pooggin, 2012; Gammon and Mello, 2015). In insects, siRNAs are also essential for the establishment of persistent viral infections (Goic et al., 2013; Lan et al., 2016a). In both hosts siRNAs based antiviral response is triggered after dsRNAs produced during virus infections are recognized by insect Dicer (DCR) or plant DCR-like (DCL) proteins and then processed into 21–24-nt virus-derived siRNAs (vsiRNAs). Argonaute (AGO) proteins loaded with one strand of the sRNA duplex associate with other proteins giving rise to RNA-induced silencing complexes that recognize and target complementary viral RNAs to their specific inactivation. In plants, fungi, and worms, RNA-directed RNA-polymerases (RDRs) use these cleaved transcripts as templates to synthesize long dsRNAs that are diced into secondary siRNAs enabling the amplification of the silencing response (Wang et al., 2010). Apart from worms, no RDRs have been found so far in animals (Zong et al., 2009). The piwi-interacting RNA (piRNAs) pathway, another sRNA-based mechanism only present in animals, was proposed to be involved in antiviral defense in mosquitoes (Morazzani et al., 2012; Miesen et al., 2016) but curiously not in adult flies (Petit et al., 2016).

Plant reoviruses may have originated from an ancestral insect virus that later in time acquired the ability to multiply in plants (Nault and Ammar, 1989). Due to frequent host alternation, virus encounters different defense strategies with unique features. In this work, we comparatively analyzed endogenous and viral-derived sRNAs in MRCV-infected *Triticum aestivum* and the planthopper vector *D. kuscheli*. In addition, we analyzed the participation of the piRNAs pathway upon infection.

## Results

### Analysis of Total sRNAs and vsiRNAs in MRCV-Infected *D. kuscheli* Insect Vector and Wheat Plants

**FIGURE 1:**
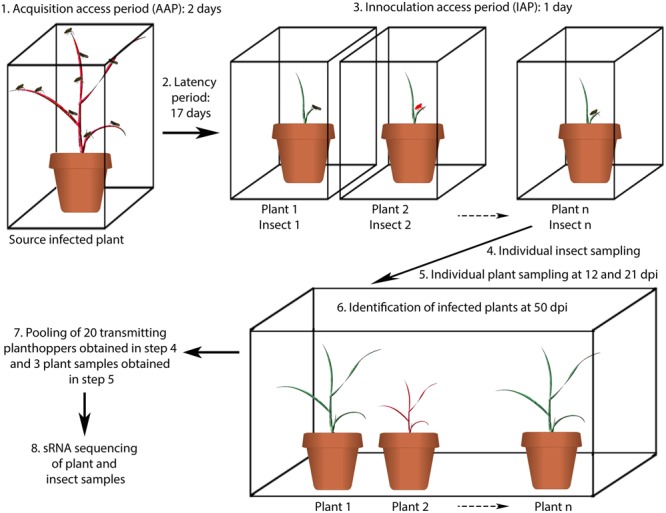
**Schematic representation of the experimental design for sRNA analysis of planthoppers and plants infected with MRCV.** Step 1: 500 *D. kuscheli* nymphs were allowed to feed on a single MRCV-infected wheat plant for 48 h. Step 2: the insects were moved to chambers containing non-infected wheat plants for 17 days (latency period). During this period, upon sap ingestion, MRCV enters and multiplies in the planthopper midgut epithelial cells until reaching a certain threshold, disseminates into midgut muscles cells, hemolymph and eventually reaches the salivary glands and the insect becomes infective. Step 3: 1:1 infection of 165 wheat seedlings in individual cages. Steps 4 and 5: individual insect and plant (young systemic leaves) samplings. Step 6: infected plants were identified by the observation of viral symptoms and enzyme-linked immunosorbent assay (ELISA) tests followed by absolute RT-qPCR analysis to measure virus RNA titters. Individual transmitting planthoppers were also identified based on infected plants. Step 7: pooling of samples. Step 8: insect and plant sRNAs extraction and sequencing. Steps 1–3 were performed in growing chambers. Step 6 was performed in a greenhouse with controlled light and temperature conditions. The experiment was repeated twice.

**FIGURE 2:**
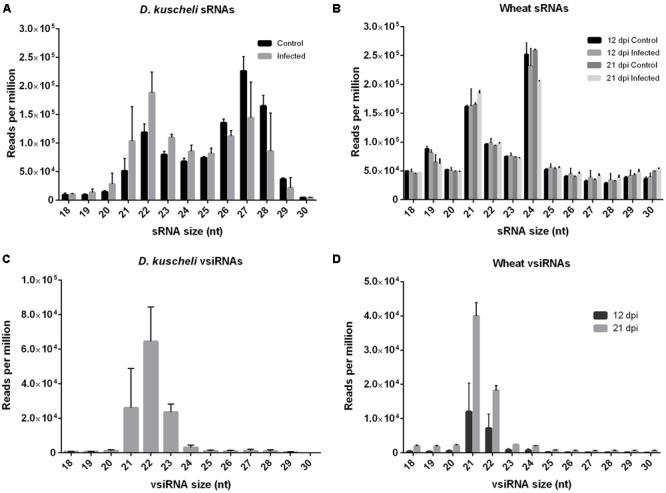
**Size distribution of total sRNAs and vsiRNAs in planthopper and wheat hosts.** Total *D. kuscheli* **(A)** and wheat **(B)** sRNAs after MRCV infection. *D. kuscheli* **(C)** and wheat **(D)** vsiRNAs after MRCV infection. Control insects were fed in non-infected plants. Control plants were treated with non-viruliferous planthoppers. Insect sRNA samples were analyzed at 19 days post-acquisition (dpa). Wheat sRNA samples were analyzed at 12 and 21 days post-infection (dpi). Reads are redundant and normalized (reads per million). Error bars: SD.

Controlled infection experiments were performed to comparatively assess the impact of MRCV infection in sRNAs profiles in wheat and insect natural hosts. The experimental design is schematized in **Figure 1**. Next, we sequenced sRNA libraries from virus-infected wheat at 12 and 21 days post-infection (dpi) and from infective *D. kuscheli* at 19 dpa with two biological replicates *per* treatment. As controls, we included wheat plants treated with non-viruliferous planthoppers and planthoppers fed on non-infected plants. After filtering adaptors and low-quality sequences, all libraries contained between 13 and 41 million reads. Next, we filtered the tRNA and rRNA-derived sequences and grouped the remaining reads according to their sizes. *D. kuscheli* libraries displayed a bimodal distribution of total sRNA reads, with one peak of 21–23-nt sRNAs and a second peak of 26–28-nt sRNAs (**Figure 2A**). The first peak may account for DCR2 activity in planthoppers (Chen et al., 2012; Li et al., 2013), whereas the second is most likely the result of the piRNA pathway (Miesen et al., 2016). Wheat libraries showed peaks at 21 and 24-nt (**Figure 2B**). Even if there is no information available on the specific roles of DCL proteins in wheat, this is the expected distribution after DCL4, DCL2, and DCL3 activities described in *Arabidopsis* and rice (Gasciolli et al., 2005; Bouché et al., 2006; Liu et al., 2007; Tomato et al., 2012) and is in accordance to observations in virus-infected wheat (Liu et al., 2014; Tatineni et al., 2014).

We also assessed vsiRNA composition by mapping total reads to a consensus sequence of MRCV genome and allowing zero, one, or two mismatches (Supplementary Table S1). To assure good quality mapping as well as to capture virus diversity, we performed all further analysis with data allowing up to one mismatch. Negligible number of reads mapped to MRCV genome in the control treatments (Supplementary Table S1). In *D. kuscheli*, 21-, 22-, and 23-nt vsiRNAs were the predominant size classes with a peak at 22-nt (**Figure 2C**). Most plant-derived vsiRNAs were 21- and 22-nt long (Liu et al., 2014) and their relative number increased from 12 to 21 dpi (**Figure 2D**).

### vsiRNAs Accumulate Differentially in Planthoppers and Plant Hosts and Their Density Does Not Correlate with RNA Accumulation of Viral Segments

**FIGURE 3:**
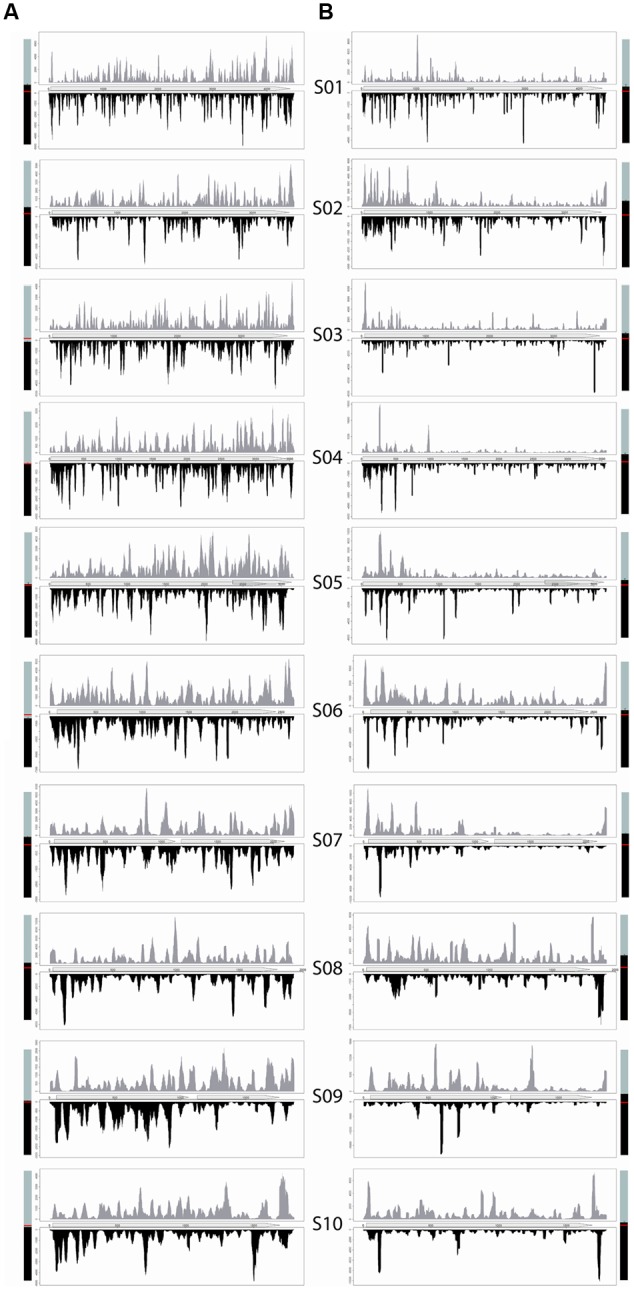
**Distribution of vsiRNAs from infected *D. kuscheli* (A)** and wheat **(B)** along the 10 dsRNAs segments of MRCV genome. Average per-base coverage of vsiRNAs is represented in the *y*-axis and the nucleotide position of MRCV genomic segments are represented across the *x*-axis. vsiRNAs identical (dark gray) or complementary (light gray) to the positive strands are displayed above and below of each segment, respectively. A schematic representation of the predicted ORFs is shown across the *x*-axis. Next to each panel, proportion of vsiRNAs reads mapping to the positive (upper) or negative (lower) strands of each segment. A red line at 50% is shown. Error bars: percent SD.

**Figure 3** displays vsiRNAs mapping profiles along MRCV genome. No strand bias was observed in any of the viral genomic segments (**Figure 3**, shown by the bars next to each profile) and vsiRNAs distribution in both hosts exhibited hot and cold spots. Although hotspots are proposed to derive from folded RNA regions (Szittya et al., 2010), we did not detect a clear correlation between hotspots and RNA structures by *in silico* secondary structure analysis (RNAfold from ViennaRNA Package; Gruber et al., 2008, data not shown). Additionally, the absence of hotspots common to both hosts appears to rule out this possibility.

The vsiRNAs profiles varied markedly between planthopper and wheat MRCV hosts. In planthoppers, vsiRNA distribution was homogeneous with hotspots evenly distributed along the segments (**Figure 3A**). In plants, the read distribution showed heterogeneous and conspicuous hotspots of vsiRNAs accumulation along each of the genome segments. Some of the peaks exhibited delayed phase mirror symmetry between strands and, with the exception of S9, most of the reads mapped to the third upstream sequences of the segments (**Figure 3B** and Supplementary Figure S1). The mapping profiles were practically identical in samples of 12 and 21 dpi (Supplementary Figure S2) but the normalized number of MRCV-derived reads was around seven times more abundant in the 21 dpi samples.

**FIGURE 4:**
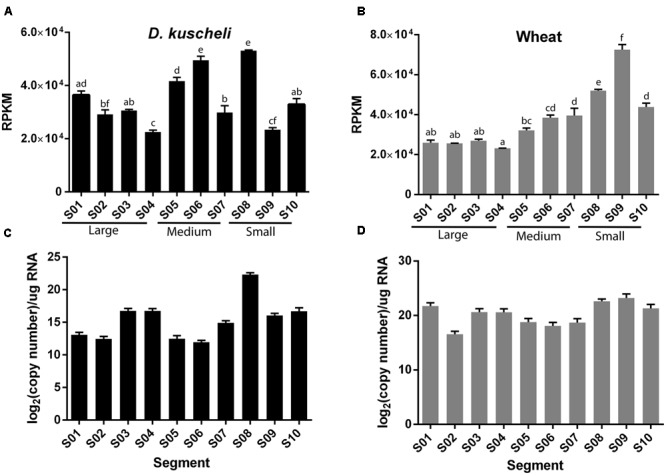
**Average vsiRNAs reads *per* kilobase *per* million reads (RPKM) values of the individual MRCV segments (S01–S10) in *D. kuscheli* (A)** and wheat **(B)**. One-way ANOVA grouping is shown with letters. Absolute quantification of the 10 MRCV genome segments (S01–S10) by RT-qPCR in *D. kuscheli* **(C)** and wheat **(D)**. Error bars: SD.

To analyze if some virus segments were preferentially targeted by RNAi machinery, we quantified the number of vsiRNAs mapping to each segment normalized by length and library size (reads *per* kilobase *per* million reads, RPKM). A one-way analysis of variance (ANOVA) test was performed and the segments were classified according to significant differences of RPKM (**Figures 4A,B**). In insects, segments S5, S6, and S8 showed higher accumulation of vsiRNAs, whereas S4 and S9 were the less densely targeted (**Figure 4A**). Interestingly, in plants, vsiRNAs density increased as segment size decreased, except in the case of S10 (**Figure 4B**). S9, which codes for the major component of the viroplasm (Maroniche et al., 2010), appears to trigger a greater silencing response in plants. In sum, these results indicate that the silencing machineries of both hosts react toward different features of the viral genome, thus giving rise to distinct vsiRNA profiles.

In reoviruses, transcription produces only positive sense single-stranded RNAs that are released to the cytoplasm from the transcriptional complexes inside the viral particles (Lourenco and Roy, 2011). Thus, vsiRNAs are the result of the antiviral response to dsRNA segments and/or to secondary structures of viral mRNAs that might be exposed to the silencing machinery (Szittya et al., 2010). To establish whether the differences in the density of vsiRNAs per segment were related to variations in viral RNA accumulation levels, we performed absolute quantitative reverse transcription PCR (RT-qPCR) of the sense strand of segments S1–S10 in insects (**Figure 4C**) and plants (**Figure 4D**). Overall, we were unable to explain the differences in normalized read counts by differences in RNA accumulation. For example, S1 was highly expressed in plants but accumulated a lower density of vsiRNAs, whereas S6 was poorly expressed in insects but accumulated a higher density of vsiRNAs. These results indicate that in MRCV-infected hosts, vsiRNAs accumulation is not directly related to viral RNA accumulation and might rather be a consequence of dsRNA accessibility to the dicing machinery.

### piRNA Pathway is Active in Planthoppers But Virus-Derived piRNAs against MRCV Were Not Detected

**FIGURE 5:**
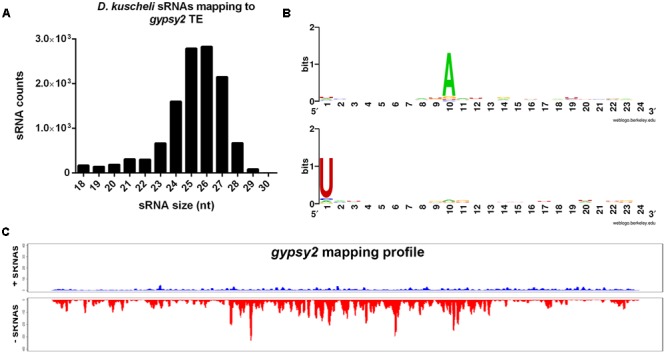
**Identification of piRNAs in *D. kuscheli*.** Size distribution of *D. kuscheli* sRNAs mapping to *Drosophila gypsy 2* TE **(A)**; sequence logo showing ping-pong amplification loop signature of piRNAs of 10 nt overlapped reads mapping to *Drosophila gypsy 2* sense (**B**, upper panel) or antisense (**B**, lower panel) TE; coverage graph of sRNAs mapping to sense (up) and antisense (low) strands of *Drosophila gypsy 2* TE **(C)**.

The piRNA pathway has been recently implicated in antiviral defense in insects (Morazzani et al., 2012; Miesen et al., 2016). However, we were unable to detect 26–28-nt sRNAs mapping to MRCV genome in infected planthoppers (**Figure 2C**). We then assessed if piRNA pathway is present and active in *D. kuscheli*. Since there are no data available of *D. kuscheli* transposable elements (TEs), we then mapped total sRNAs to a *Drosophila* TE database obtained from FlyBase v.FB2016_05 (Attrill et al., 2015). On average, 3.97% of the reads mapped to TEs in the database. Out of the 80 transposable elements with more than 2000 mapping reads, 61 showed clear evidence of being targeted by piRNAs. This is shown by the size of the sRNAs and a sequence logo of 10-nt overlapping reads with a ping-pong signature. As an example, **Figure 5** displays the results for *Drosophila melanogaster gypsy2* transposon. Indeed, 11,902 sRNAs predominantly 24–27-nt long (**Figure 5A**) mapped almost exclusively to the antisense strand of *gypsy2* (**Figure 5B**). A strong bias of A in the 10th position of the sense strand and U in the first position of the antisense strand was evident (**Figure 5C**). Overall, these results indicate that the piRNA pathway is active in planthoppers but unlike what has been found in other virus-infected insects, we did not observe anti-MRCV piRNAs.

## Discussion

In insects, antiviral RNA silencing limits virus accumulation and this process may lead to persistence (Lan et al., 2016b) and can affect vector competence (Lan et al., 2016a) and transmission efficiency (Argüello Caro et al., 2013; Matsukura et al., 2015). Researchers have extensively studied siRNA pathway in *D. melanogaster* and mosquitoes (Bronkhorst and Van Rij, 2014; Xu and Cherry, 2014) and to a lesser extent in leafhopper and planthopper vectors that transmit persistent-propagative viruses (Li et al., 2013; Lan et al., 2016b). In most of these insects, DCR2 predominantly gives rise to 21-nt vsiRNAs that limit virus infection (Galiana-Arnoux et al., 2006; Van Rij et al., 2006; Wang et al., 2006; Schnettler et al., 2013; Sekhar Nandety et al., 2013; Lan et al., 2016b). However, the results presented here indicate that for *D. kuscheli* the 22-nt species is the most abundant followed by the 21- and 23-nt long vsiRNAs (**Figure 2**), in agreement with previous findings in other planthopper species (Chen et al., 2012; Xu Y. et al., 2012, 2014; Li et al., 2013, 2014; Lan et al., 2016a). Interestingly, DCR2 from the brown planthopper *Nilaparvata lugens* (Hemiptera: *Delphacidae*) lacks the carboxy-terminal dsRNA binding domain (dRBD) that is present in *Drosophila* and other insects (Zhang X.-Y. et al., 2013). This difference could account for differences in the molecular ruler that determines the sRNA length.

Insects and other animals produce piRNAs involved in maintaining genome stability in germ line cells by targeting transposons (Halic and Moazed, 2009). Interestingly, emerging functions have been recently proposed for piRNAs (Czech and Hannon, 2016; Sarkar et al., 2017) including their participation in antiviral defense in mosquitoes (Morazzani et al., 2012; Miesen et al., 2016). In fact, Lan et al. (2016b) detected piRNAs in leafhoppers, whereas Xu et al. (2013) showed that the planthopper *N. lugens* codes for piRNA pathway core components including AGO 3, Piwi and Aubergine. Our study allowed the detection of piRNAs in planthoppers for the first time but failed to detect virus-derived piRNAs of 24–26-nt (**Figure 5C**). Curiously, piRNA production or turnover seems affected by MRCV infection. Even though the decrease shown in **Figure 2A** is not statistically significant, the role of infection in the control of transposons and host gene regulation deserves further studies.

The model plant *Arabidopsis* codes for 4 DCLs, 6 RDRs, and 10 AGOs, whereas wheat and other monocots code for 5 DCL proteins (Margis et al., 2006), 5 RDRs [RDR1, RDR2, RDR3a, RDR3b, RDR6 (Zong et al., 2009), and possibly 19 AGOs (Kapoor et al., 2008)]. DCL4, DCL2, and DCL3 are involved in processing viral RNAs giving rise to vsiRNAs of 21-, 22-, and 24-nt respectively. Parent et al. (2015) have established hierarchical roles for DCL4 and DCL2 and reported that the 21-nt vsiRNAs is the most abundant class followed by 22-nt vsiRNAs.

Small differences in the quality or quantity of the starting samples can affect the outcome of sRNA analysis. For this reason, the accumulation of defined species of sRNAs can be compared within a sample but not between samples. In this sense, 21/24 sRNAs ratios are a useful parameter to understand the global impact of infection on the biogenesis of sRNAs. Interestingly, wheat MRCV infection in leaves yielded a 0.9 21-/24-nt ratio at 21 dpi, due to a slight increase in 21-nt species and a slight decrease in the 24-nt species upon infection. In turn, control plants displayed a ∼0.6 21-/24-nt ratio. Along the same line, infections with a phloem-limited rice virus (Rajeswaran et al., 2014) and a wheat virus (Tatineni et al., 2014) displayed similar slight changes in 21- and 24-nt species abundance. These results contrast to what was reported in virus infections of dicotyledonous plants where 21-/24-nt ratios are much higher (Donaire et al., 2009; Herranz et al., 2015).

Our results showed vsiRNAs of both polarities in even proportions (practically 50% of the reads for each segment; **Figure 3**) in plant and insect hosts. Furthermore, these vsiRNAs mapped to the entire genome. These findings suggest that the templates for vsiRNAs production are full-length viral dsRNAs. In plants, the activity of RDRs can account for the antisense vsiRNAs. However, finding vsiRNAs derived from the negative strand in *D. kuscheli* was somehow surprising since in animal reoviruses negative strands are synthesized within the preassembled cores protected from the silencing machinery (Lourenco and Roy, 2011). Moreover, no RDRs have been so far detected in insects (Zong et al., 2009). These findings are in line with studies in insects infected with other members of the family *Reoviridae* such as the leafhopper *Homalodisca vitripennis* (Sekhar Nandety et al., 2013), the small brown planthopper (*Laodelphax striatellus*) (Li et al., 2013), *Bombyx mori* (Zografidis et al., 2015), and for *Culicoides sonorensis*-derived cells (Schnettler et al., 2013).

So, how does the silencing machinery has access to viral negative RNAs in insects? In animal reoviruses, virus assembly is coupled with genome replication in a highly regulated process. Rotavirus plus strand RNAs are selectively packaged into assembling cores and the negative strands are synthesized only after the structure of the virus polymerase is modified by interaction with the major component of the core (Trask et al., 2012; Gridley and Patton, 2014). A partial uncoupling of genome replication and assembly could expose dsRNA to the silencing machinery. Alternatively, inter segment complementarity prior to the encapsidation could be the trigger for vsiRNAs production (McDonald et al., 2016). This is supported by the study of Weber et al. (2006) in which they detected dsRNA in cells infected with a mammalian orthoreovirus.

Another hypothesis to explain the presence of vsiRNAs derived from the negative strand in insects is that parts of MRCV genome are somehow integrated into the planthopper genome after the infection (Liu et al., 2010). The transcription of such integrated sequences may give rise to viral dsRNA that would be recognized and processed by the RNAi machinery producing vsiRNAs. In *Drosophila*, endogenous reverse transcriptases convert viral RNA to DNA forms that produce dsRNAs upon transcription. In turn, these dsRNAs are processed giving rise to vsiRNAs that partially suppress virus replication contributing to the establishment of a persistent infection (Goic et al., 2013).

An alternative possibility is that MRCV-derived endogenous viral elements (EVEs) already exist integrated in planthopper genomes. Such elements, many of them derived from viruses with no DNA stage, are present in insect genomes (Drezen et al., 2016), such as the brown planthopper nudivirus EVEs (Cheng et al., 2014).

When we analyzed the distribution of vsiRNAs along the virus genome (**Figure 4**), we identified hotspots in both hosts and in the sense and antisense strands, particularly within the upstream 30% of almost all virus segments (Supplementary Figure S1). This result could be partially explained by the well-known dsRNAs panhandle structures formed by interactions between reovirus 5′ and 3′ terminal ends. Alternatively, these heavily targeted regions could be explained by a decoy mechanism such as the one observed upon the infection with rice tungro bacilliform virus, where decoy dsRNA restricts siRNA production to the upstream region to protect other regions of the viral genome from the repressive action of vsiRNAs (Rajeswaran et al., 2014). We evaluated possible associations between internal hotspots and RNA secondary structures within segments but this approach did not satisfactorily explain our results (data not shown). Nevertheless, further studies using more complex models considering inter-segment complementarity should be performed to test this hypothesis.

Remarkably, only in plants, vsiRNAs density increases as segment size decreases (**Figure 4B**) and the small segments (S9, and S8, 1879 and 1931 nt, respectively) were more efficiently targeted than the medium (S7–S5, ranging in size between 3162 and 2186 nt) and the large ones (S1–S4, ranging in size between 4501 and 3566 nt). A higher abundance of these segment RNAs cannot explain this finding, as they were measured by absolute qPCR (**Figure 4D**). These differences may be reflecting the sequential packaging mechanism proposed for animal reoviruses that involves both RNA–protein and RNA–RNA interactions. This process is believed to initiate with S10 and the rest of the segments are sequentially packaged according to their size (Sung and Roy, 2014; Fajardo et al., 2015, 2016; Boyce et al., 2016). Therefore, the higher density of vsiRNAs toward the smaller segments may reflect different access of the silencing machinery toward the virus genome segments while they are engaged in the formation of supramolecular RNA complexes through RNA–RNA interactions driven by base pairing immediately prior to packaging.

Altogether, our work reflects differences in patterns of sRNAs from a snapshot sampling of different hosts in response to a segmented dsRNA virus. Further understanding of the underlying silencing mechanisms is necessary to improve the biotechnological use of RNAi as an antiviral strategy both in plants and in insects.

## Materials and Methods

### Source and Maintenance of Insects and Virus

The *D. kuscheli* colony used in this study was obtained and reared under artificial conditions since 2008 at the Vector’s Laboratory of IPAVE-CIAP (INTA, Argentina). The MRCV isolate used as the viral inoculum was obtained from infected oat plants collected in 2008 in Río Cuarto, Córdoba Province, Argentina, and maintained in wheat (*T. aestivum* cv. ProINTA Federal) by consecutive transmissions using *D. kuscheli* as previously described by Truol et al. (2001).

### Transmission Trials to Obtain MRCV-Infected Material

Transmission trials were carried out using wheat as host (Truol et al., 2001) as described in **Figure 1**. Groups of male and female *D. kuscheli* planthoppers were allowed to reproduce on healthy wheat plants in plastic containers. Twenty-four hours after oviposition, adults were removed and the plants were grown in breeding chambers under controlled conditions of temperature (24 ± 3°C), humidity (50%), and photoperiod (16 h light, 8 h dark) for egg development. Second instar nymphs were obtained 6 days after hatching, and used for individual transmission assays. At least 500 nymphs were allowed to feed on MRCV-infected wheat or in non-infected wheat (as a control) for 48 h (acquisition access period—AAP). The insects were then moved to chambers containing non-infected wheat plants for 17 days (latency period). Next, 1:1 transmission assays were performed by individually transferring one insect to a single non-infected wheat seedling cv Pro INTA Federal (Truol et al., 2001) (inoculation access period—IAP). After 24 h, planthoppers were individually placed in 1.5 ml microtubes in liquid nitrogen and stored at -80°C. Finally, the plants were conditioned in a greenhouse with temperature controlled conditions and daily irrigation. The plants were rotated regularly within the greenhouse to reduce any positional effects. Twelve and 21 days after IAP, the leaf previous to the flag leaf was collected, frozen in liquid nitrogen and placed at -80°C. MRCV symptoms appeared near 30 days after IAP (Truol et al., 2001). Then, the wheat plants were individually identified as MRCV symptomatic or non-symptomatic and MRCV infection was confirmed by double antibody sandwich ELISA (DAS-ELISA) at 50 dpi as in Truol et al. (2001). Insects were classified as transmitting or non-transmitting, according to whether they were able to inoculate MRCV to wheat seedlings. The experiments were performed with two replicates of 165 insects each.

### Small RNA Sequencing and Mapping to MRCV Genome

Total RNA from the younger fully expanded leaf of wheat plants or whole insect pools were extracted using mirVana (Thermo Fisher Scientific Inc.) according to the manufacturer’s instructions. RNA integrity was verified using a Bioanalyzer 2100 RNA chip (Agilent Technologies). Then, 18–30-nt sRNAs were excised from sodium dodecyl sulfate polyacrylamide gel electrophoresis (SDS-PAGE) gels, purified, used for sRNA library preparation and finally sequenced using Illumina technology. Low quality reads and adaptor contaminants were clipped using Sickle and Scythe (Joshi and Fass, 2011; Buffalo, 2014) and mapped to MRCV genome (GenBank Accession numbers: NC_008733, NC_008730, NC_008732, AF395873, NC_008735, NC_008731, NC_008736, AF395872, NC_008737, and NC_008734) using Burrows-Wheeler Aligner (BWA) (Li and Durbin, 2009). Consensus genome was built using samtools and bcftools (Li et al., 2009) and then reads were remapped to these sequences with BWA allowing zero, one, or two mismatches. Unless other stated, read numbers were scaled to “reads per million” (rpm) based on the total sRNA read numbers of the corresponding library and average values of the two biological replicates. Raw sequences were deposited in NCBI Sequence Read Archive (SRA)^1^ under the accession numbers SRR5270350, SRR5270349, SRR5270348, SRR5270347, SRR5270346, SRR5270345, SRR5270344, SRR5270343, SRR5270448, SRR5270447, SRR5270446, and SRR5270445.

### Analysis of *D. kuscheli* TEs

*Delphacodes kuscheli* reads were mapped to *D. melanogaster* TE database from FlyBase v.FB2016_05^2^ allowing one mismatch. Only TEs with more than 2000 reads were kept for further analysis. Then, size histograms of mapped reads were built for each TE and 10-nt-overlapping reads were used for sequence logo construction. Genome coordinates and sequences of mapped reads were extracted using samtools and sequence logo was constructed using custom R scripts and WebLogo (Crooks et al., 2004).

### Mapping Density Analyses

Average *per*-base coverage plots were built using bedtools genomecov algorithm (Quinlan and Hall, 2010) and custom R scripts. The number of reads mapping to each segment and the number of reads mapping to each tenth part of each segment was extracted using samtools and normalized according to the library size.

### Absolute qPCR

Primer sequences (Supplementary Table S2) were designed using Primer3 software (Untergasser et al., 2012). Different plasmids containing complete or partial sequences of MRCV segments were used for the construction of external standard curves for absolute quantification as previously described (Argüello Caro et al., 2013). Synthesis of cDNA was carried out from 1 μg of DNaseI-treated total RNA by using Superscript III (Thermo Fisher Scientific Inc.) and random primers, according to the manufacturer’s protocol. qPCR reactions were carried out in an ABI7500 Real Time System (Applied Biosystems) using a Fast SYBR Green Master Mix (Thermo Fisher Scientific Inc.). Each 20-μL reaction was comprised of 10 μL 2× Fast SYBR Green Master Mix, 0.5 μL of forward and reverse primers (10 μM each), 8 μL distilled, deionized H_2_O, and 1 μL of a 10-fold dilution of cDNA. *D. kuscheli* and wheat reactions were carried out with three and six biological replicates, respectively. Reference genes Dk-UBI for planthoppers (Maroniche et al., 2011) and Ta-GTPB for wheat (Zhang K. et al., 2013) were used as internal controls for normalization.

## Author Contributions

LdH, AD, MM, VM, M-CS, SA, and MdV designed the study, LdH conducted all bioinformatics analysis and performed sample processing and qPCR experiments, LdH, DZ, VM, M-CS, SA, and MdV analyzed and interpreted data, LdH, AD, MM, EA, GL, and GT, performed the transmission experiment and sampled the material, DZ contributed with bioinformatics assistance, LdH and MdV wrote the manuscript. HB prepared the *D. kuscheli* libraries and obtained the sRNA sequences. All authors read and approved the final manuscript.

## Conflict of Interest Statement

The authors declare that the research was conducted in the absence of any commercial or financial relationships that could be construed as a potential conflict of interest.

## Footnotes

1 http://www.ncbi.nlm.nih.gov/sra

2 http://flybase.org/static_pages/docs/datafiles.html#transposon_sequence_set
