# Supplementary material for: Mal de Río Cuarto Virus Infection Triggers the Production of Distinctive Viral-Derived siRNA Profiles in Wheat and Its Planthopper Vector
Source: Front Plant Sci. 2017 May 10;8:766. doi: 10.3389/fpls.2017.00766 (PMC5423983; doi:10.3389/fpls.2017.00766)
Supplement: Supplementary file 1 [file Table_1.PDF]

Supplementary Material

Mal de Río Cuarto virus infection triggers the production of distinctive viral-derived siRNAs profiles in wheat and its planthopper vector

Luis Alejandro de Haro, Analía Delina Dumón, María Fernanda Mattio, Evangelina Beatriz Argüello Caro, Gabriela Llauger, Diego Zavallo, Hervé Blanc, Vanesa Claudia Mongelli, Graciela Truol, María-Carla Saleh, Sebastián Asurmendi, Mariana del Vas*

***Correspondence:**
Mariana del Vas: delvas.mariana@inta.gov.ar

1 Supplementary Table S1.

Mapping rates (as percentage of total reads in each library) to MRCV genome for each of the small RNA libraries used in this study. dpa: days post-acquisition; dpi: days post-infection. C: Control treatment, I: MRCV-infected treatment. The experiment was repeated twice (R1 and R2).

**Host**	**Time point**	**Rep**	**No mismatch**		**up to 1 mismatch**		**up to 2 mismatches**	
			**C**	**I**	**C**	**I**	**C**	**I**
D. kuscheli	**21 dpa**	**R1**	0.00%	5.52%	0.01%	8.52%	0.01%	9.14%
		**R2**	0.00%	8.84%	0.01%	11.87%	0.01%	12.43%
**Wheat**	**12 dpi**	**R1**	0.00%	2.04%	0.01%	2.29%	0.31%	2.62%
		**R2**	0.00%	0.77%	0.01%	0.87%	0.26%	1.11%
	**21 dpi**	**R1**	0.00%	4.63%	0.01%	5.26%	0.14%	5.66%
		**R2**	0.00%	3.96%	0.00%	4.53%	0.15%	4.92%
