# Supplementary material for: Mal de Río Cuarto Virus Infection Triggers the Production of Distinctive Viral-Derived siRNA Profiles in Wheat and Its Planthopper Vector
Source: Front Plant Sci. 2017 May 10;8:766. doi: 10.3389/fpls.2017.00766 (PMC5423983; doi:10.3389/fpls.2017.00766)
Supplement: Supplementary file 2 [file Table_2.PDF]

Supplementary Material

Mal de Río Cuarto virus infection triggers the production of distinctive viral-derived siRNAs profiles in wheat and its planthopper vector

Luis Alejandro de Haro, Analía Delina Dumón, María Fernanda Mattio, Evangelina Beatriz Argüello Caro, Gabriela Llauger, Diego Zavallo, Hervé Blanc, Vanesa Claudia Mongelli, Graciela Truol, María-Carla Saleh, Sebastián Asurmendi, Mariana del Vas*

***Correspondence:**
Mariana del Vas: delvas.mariana@inta.gov.ar

1 Supplementary Table S2.

Sequence of the primers used in the qPCR analysis of Figure 6 in every segment of MRCV genome.

Primer name	Oligo Sequence (5' -> 3')
qMRCV-S1_F	TCGTCGTGCGAGAGTAATTG
qMRCV-S1_R	AGGATACATCTCCGGTCGTG
qMRCV-S2_F	CTGGATTCCCCTCATTTCAA
qMRCV-S2_R	ATGTTGTCAGGTGTGCCAAA
qMRCV-S3_F	AGGCCACCTCAAACAATCAG
qMRCV-S3_R	ATTGTTGGGCACTGTCATCA
qMRCV-S4_F	GAGAAAATTCCAACCGACGA
qMRCV-S4_R	ACACTGGGGAATTCAACTGC
qMRCV-S5_F	CACGAAGGTTCATCCCTGTT
qMRCV-S5_R	TGCAAGTCAGCGATTTGAAC
qMRCV-S6_F	AAACTAAGCGCCAAGAGCAA
qMRCV-S6_R	CGCTGTTCTCTCAACCATGA
qMRCV-S7_F	GTTTGGCAACTCCTCAGCTC
qMRCV-S7_R	ATAAAAGCGTCACCCATTGC
qMRCV-S8_F	GCAGACAAGCATGAGATGGA
qMRCV-S8_R	GTGATGTGTTCGGTGCAATC
qMRCV-S9_F	GACGGCATTTTTGACCTGAT
qMRCV-S9_R	TCACGCTCAAGTGTTGGAAG
qMRCV-S10_F	AACGGAACAAGTCGAAATGG
qMRCV-S10_R	AGTACGTCGCCGATTTTACG
