# Supplementary material for: Mal de Río Cuarto Virus Infection Triggers the Production of Distinctive Viral-Derived siRNA Profiles in Wheat and Its Planthopper Vector
Source: Front Plant Sci. 2017 May 10;8:766. doi: 10.3389/fpls.2017.00766 (PMC5423983; doi:10.3389/fpls.2017.00766)
Supplement: Supplementary file 3 [file Image_1.PDF]

Supplementary Material

Mal de Río Cuarto virus infection triggers the production of distinctive viral-derived siRNAs profiles in wheat and its planthopper vector

Luis Alejandro de Haro, Analía Delina Dumón, María Fernanda Mattio, Evangelina Beatriz Argüello Caro, Gabriela Llauger, Diego Zavallo, Hervé Blanc, Vanesa Claudia Mongelli, Graciela Truol, María-Carla Saleh, Sebastián Asurmendi, Mariana del Vas*

***Correspondence:**
Mariana del Vas: delvas.mariana@inta.gov.ar

1 Supplementary Figure S1

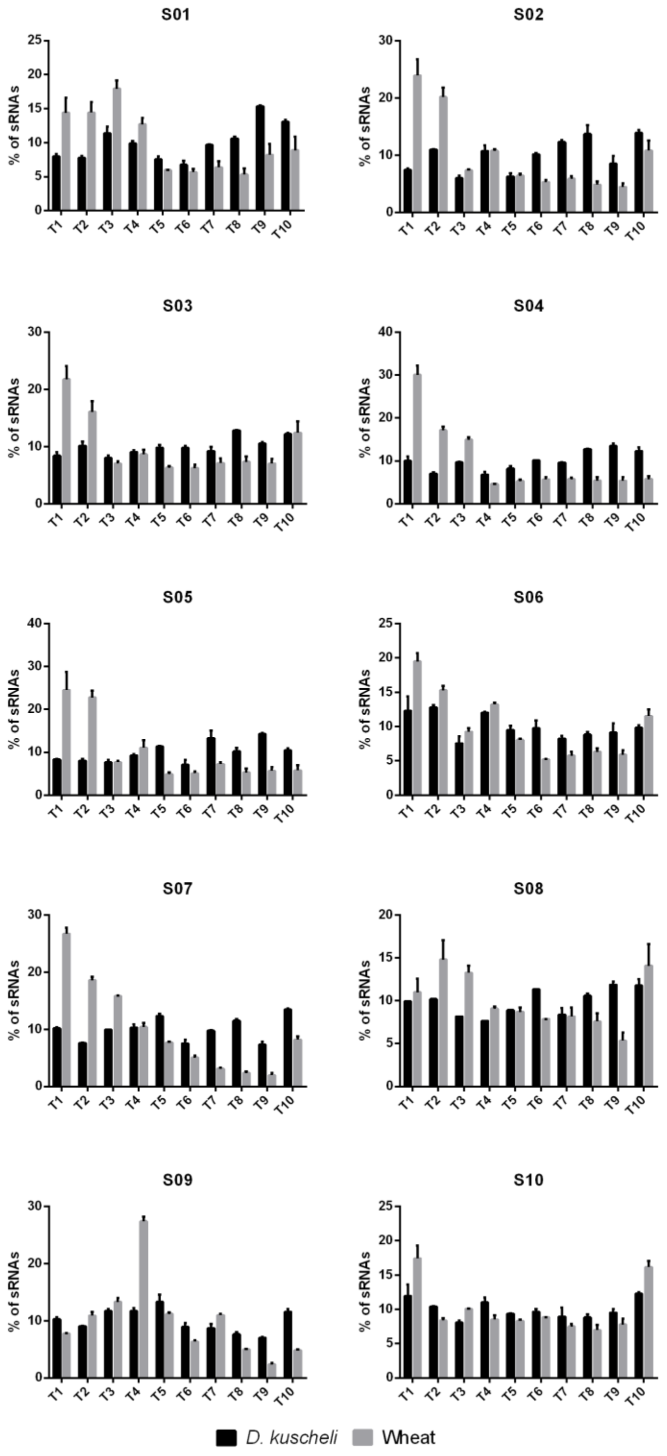

Supplementary Figure S1. Distribution of vsiRNAs from *D. kuscheli* and wheat within each MRCV genome segment (S1 to S10). All MRCV genome segments were divided in ten equal fractions (T1 to T10). Percentage bar graphs represent the mean number of total vsiRNAs that mapped to the ten fractions within each segment.
