# Supplementary material for: Mal de Río Cuarto Virus Infection Triggers the Production of Distinctive Viral-Derived siRNA Profiles in Wheat and Its Planthopper Vector
Source: Front Plant Sci. 2017 May 10;8:766. doi: 10.3389/fpls.2017.00766 (PMC5423983; doi:10.3389/fpls.2017.00766)
Supplement: Supplementary file 4 [file Image_2.PDF]

Supplementary Material

Mal de Río Cuarto virus infection triggers the production of distinctive viral-derived siRNAs profiles in wheat and its planthopper vector

Luis Alejandro de Haro, Analía Delina Dumón, María Fernanda Mattio, Evangelina Beatriz Argüello Caro, Gabriela Llauger, Diego Zavallo, Hervé Blanc, Vanesa Claudia Mongelli, Graciela Truol, María-Carla Saleh, Sebastián Asurmendi, Mariana del Vas*

***Correspondence:**
Mariana del Vas: delvas.mariana@inta.gov.ar

1 Supplementary Figure S2

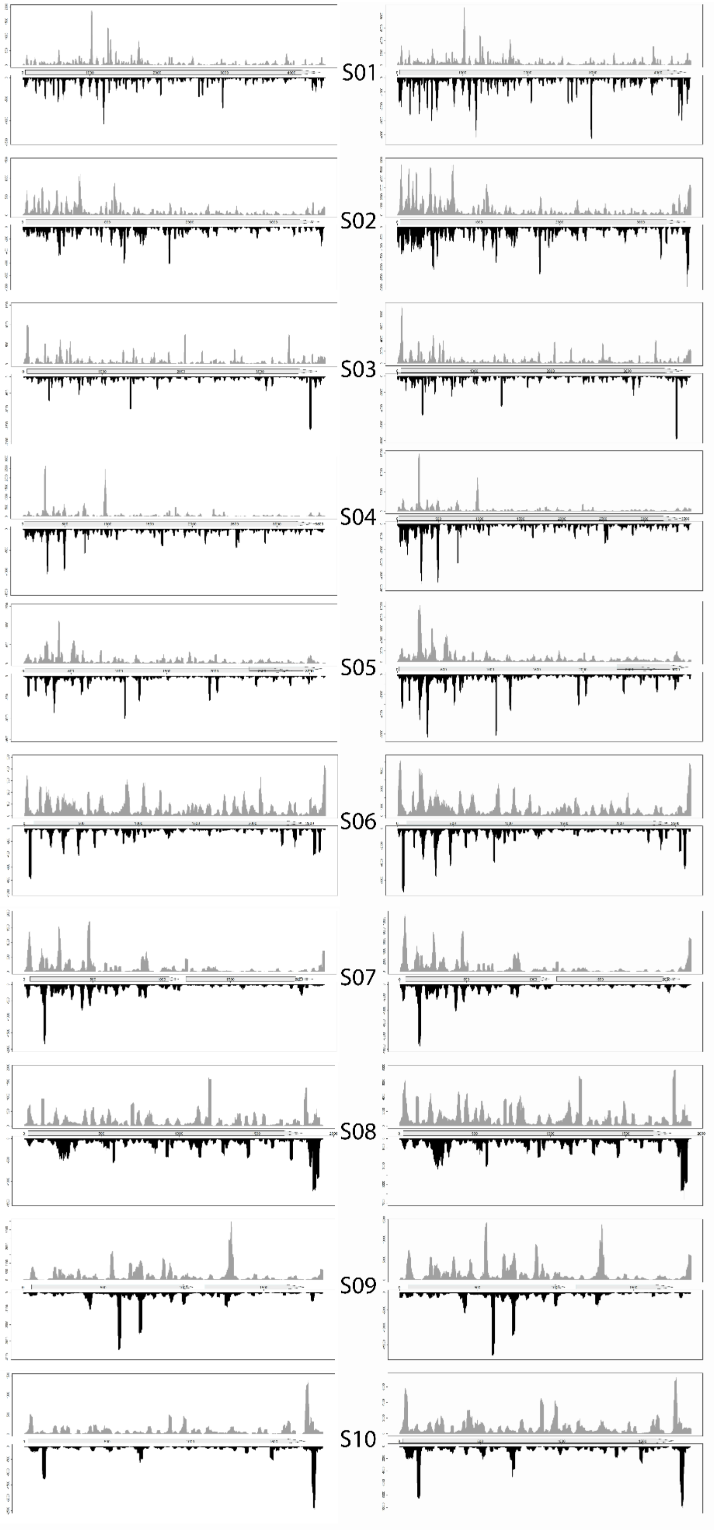

.

Supplementary Figure S2. Distribution of vsiRNAs from MRCV infected wheat at 12 dpi (left panel) and 21 dpi (right panel) along the ten dsRNAs genome segments (S1 to S10). Average per-base coverage of vsiRNAs is represented in the Y-axis and the nucleotide position of MRCV genomic segments are represented across the X-axis. vsiRNAs identical or complementary to the positive strands are displayed above and below of each segment respectively. A schematic representation of the predicted ORFs is shown across the X-axis.
